# Progesterone improved the behavior of PC12 cells under OGD/R by reducing FABP5 expression and inhibiting TLR4/NF-κB signaling pathway

**DOI:** 10.1007/s10863-023-09998-z

**Published:** 2023-12-18

**Authors:** Chunlin Li, Bowen Li, Linglong Qu, Ruichang Song, Hui Liu, Shanshan Su

**Affiliations:** 1 Department of Neurology, Affiliated Hospital of Shandong University of Traditional Chinese Medicine, No. 16369 Jing Shi Road, Lixia District, Jinan, Shandong 250014 People’s Republic of China; 2 Department of Traditional Chinese Medicine, College of Traditional Chinese Medicine, Shandong University of Traditional Chinese Medicine, No. 16369 Jing Shi Road, Lixia District, Jinan, Shandong 250014 People’s Republic of China; 3 Department of Chinese Internal Medicine, First School of Clinical Medicine, Shandong University of Traditional Chinese Medicine, No. 16369 Jing Shi Road, Lixia District, Jinan, Shandong 250014 People’s Republic of China; 4 Department of Nephrology, Affiliated Hospital of Shandong University of Traditional Chinese Medicine, No. 16369 Jingshi Road, Lixia District, Jinan, Shandong 250014 People’s Republic of China

**Keywords:** FABP5, Oxygen glucose deprivation/reperfusion, Progesterone, PC12 cells

## Abstract

Herein, PC12 cells were applied to detect the impact of progesterone under oxygen glucose deprivation/reperfusion (OGD/R) stimulation. The cell proliferation of PC12 cells was evaluated by cell counting kit-8 assay, and the concentrations of MDA, ROS and SOD were examined by their corresponding Enzyme Linked Immunosorbent Assay kits. The invasion and migration properties of PC12 cells were evaluated by transwell and wound healing assays, respectively. The expression patterns of related genes were evaluated by western blot and qPCR. Under OGD/R stimulation, progesterone treatment could elevate the viability of PC12 cells, reduce the levels of MDA and ROS, and elevate the concentration of SOD. Moreover, progesterone treatment could strengthen the invasion and migration abilities of PC12 cells under OGD/R condition, as well as decrease the apoptosis and inflammation. FABP5 expression was significantly increased in PC12 cells under OGD/R stimulation, which was reversed after progesterone stimulation. Under OGD/R stimulation, the protective effects of progesterone on PC12 cells were strengthened after si-FABP5 treatment. The protein levels of TLR4, p-P65 NF-κB, and P65 NF-κB in OGD/R-induced PC12 cells were increased, which were inhibited after progesterone treatment. Progesterone exerted protective effects on PC12 cells by targeting FABP5 under OGD/R stimulation.

**Supplementary Information:**

The online version contains supplementary material available at 10.1007/s10863-023-09998-z.

## Introduction

The main manifestation of ischemic stroke is cerebral hypoxemia caused by vascular obstruction (Yan et al. 2019). Apart from ischemia, reperfusion after ischemia can also damage the brain, such as inflammation, oxidative stress, etc. (Li et al. 2020). In the past, the main treatments for stroke have focused on neuroprotection, saving ischemic neurons from irreversible damage (Zhou et al. 2010). However, significant benefits was not observed in clinical stroke trials (Han et al. 2018). The occurrence of ischemic stroke is often accompanied by changes in gene expression and protein levels, which makes it possible to investigate the therapeutic mechanism of drugs at the molecular level.

Progesterone is a primary gonadal hormone produced mainly by the ovaries of female and the testes and adrenal cortex of male (Singh and Su 2013). In addition to regulating gene expression, progesterone could perform its function through the activation of signal transduction pathways (Singh 2001). Progesterone could perform beneficial effects in various experimental models that simulate actual pathogenic aspects of brain dysfunction. For example, physiologically related progesterone concentrations have been presented to obviously mitigate glutamate-induced oxidative damage and glucose deprivation-caused neuronal injury (Kaur et al. 2007), moreover, progesterone could also protect against amyloid β-peptide-caused toxicity in primary hippocampal cultures (Goodman et al. 1996). In addition, progesterone has also been found to be an effective neuroprotective agent in animal models of stroke. A previous study demonstrated that the addition of progesterone before middle cerebral artery occlusion contributed to an obvious reduction in cerebral infarction and lesser injury (Jiang et al. 1996). Significantly, post-ischemic administration of progesterone has also been found to be protective (Moralí et al. 2005). However, the specific mechanism of progesterone’s neuroprotective effect has not been clearly elucidated.

Fatty acid-binding proteins (FABPs) are existed in numerous tissues and perform important functions in the pathogenesis of various primary diseases (Amiri et al. 2018). Most mammals generate 12 different FABP subtypes, three of which are produced in the brain, including FABP5 (Shimamoto et al. 2015; Liu et al. 2010). Moreover, a recent study reported that FABP5 could elevate infract volumes and the suppressing FABPs could ameliorate the ischemic damage (Guo and Kawahata 2021). Information from Comparative Toxicogenomics Database (CTD) website suggested that FABP5 was regarded as a potential target of progesterone, and FABP5 pattern was suppressed by progesterone. Herein, PC12 cells stimulated by oxygen glucose deprivation/reperfusion (OGD/R) were used to mimic the neuronal injury to detect the effect of progesterone/FABP5 on neuronal injury.

## Materials and methods

### Cell culture and OGD/R model establishment

Rat pheochromocytoma (PC12) cell line was purchased from Cell Bank of the Chinese Academy of Sciences (Shanghai, China) and cultivated in Dulbecco’s modified Eagles’s medium (DMEM) including 10% fetal bovine serum (FBS). Cultivation was maintained in a humidified incubator composed of 95% air and 5% CO_2_ at 37 °C. PC12 cells were subjected to OGD/R condition to simulate ischemic-like condition. The specific operation steps were described below: DMEM was replaced by Hanks Balanced Salt (without glucose), then the culture condition was changed to oxygen-deficient environment (composed of 95% nitrogen and 5% CO_2_) for 6 h. In the final stage of the OGD, the medium was changed to growth medium including 4.5 g/L glucose and cultured under normal conditions for 12 h reoxygenation. PC 12 cells without OGD/R treatment were considered as a control. Gradient concentrations of progesterone were used to stimulate PC12 cells.

### Cell proliferation assay

A Cell Counting Kit-8 (CCK-8) was employed to detect the viability of PC12 cells. In brief, PC12 cells were implanted in a 96-well plate (3000 cells/well). After treatment, CCK-8 solution (10 µL) was put into per well and cultured at 37 °C for 1.5 h. Then, the data were analyzed on a microplate reader. The optical density (OD) was read at 450 nm wavelength based on the supplier’s specification, and each group had three triplicate wells.

### Wound healing assay

The treated PC12 cells were implanted in a serum-free medium in a 6-well plate, and a 10 µL micropipette tip was applied to scratch and establish a wound each well. Then, the cells were monitored during the regrowth, and images were captured at 0 h and 48 h.

### Transwell assay

Migration of PC12 cells was evaluated using Transwell chamber with a pore size of 8 mm. In brief, 3 × 10^4^ PC12 cells per well were implanted in 24-well plates and subjected to different treatment. Next, adherent cells were harvested and washed with PBS for three times. Then, 1 × 10^4^ PC12 cells of each sample were resuspended in 200 µL culture medium excluding serum and put into the upper chamber. Complete culture medium was put into the lower chamber. After cultivation at 37 °C for 24 h, non-migratory cells in the upper chamber were removed using a cotton swab, and migratory cells in the lower chamber were counted under a microscope.

### ELISA

Concentrations of malondialdehyde (MAD), reactive oxygen species (ROS), superoxide dismutase (SOD) in the supernatant were determined by corresponding commercial ELISA kits following the supplier’s direction.

### Real-time quantitative polymerase chain reaction (qPCR)

The whole RNA was extracted by TRIzol reagent (Invitrogen, USA) basing on the supplier’s specification. Then, reverse transcription was proceed using a cDNA synthesis kit (TaKaRa, Japan). qPCR reactions were carried out by using TaKaRa SYBR Premix Ex Taq II on a PCR amplifier. The relative abundance of mRNA was calculated by using 2^−ΔΔCT^ method, and GAPDH was deemed as an internal control.

### Western blot

The whole protein was isolated from the PC12 cells by using lysis buffer including 1 mM PMSF on ice. The concentration of protein was quantified by a bicinchoninic (BCA) protein assay. Then, sodium dodecyl sulfate polyacrylamide gel electrophoresis (SDS-PAGE) was applied to isolate an equal amount of protein, and transfer box was transferred the protein to a polyvinylidene fluoride (PVDF) membrane. The membrane was sealed with 5% non-fat milk at room temperature for 1 h, and then the membrane was incubated in 5% milk in Tris-buffered saline with Tween 20 (TBST) solution including the following primary antibodies (FABP5, Bax, Bcl-2, cleaved caspase3, IL-1, IL-6, TNF-α, and GAPDH) overnight at 4 °C. After washing with TBST for three times, the membrane was incubated with the corresponding antibodies for 1 h at room temperature, and the protein bands were visualized by enhanced chemiluminescence (ECL). The grey values of protein bands were analyzed by using Image J software.

### Statistical analysis

All experimental data were obtained from at least three independent experiments and expressed as mean ± standard deviation (SD). Statistical analysis was carried out by ANOVA using GraphPad Prism 8.0 software, followed by Bonferrion’s post hoc test. Differences were taken as significant if the probability of the difference occurring by chance was < 0.05.

## Results

### The protective effects of progesterone on PC12 cells have been revealed under OGD/R condition

**Fig. 1:**
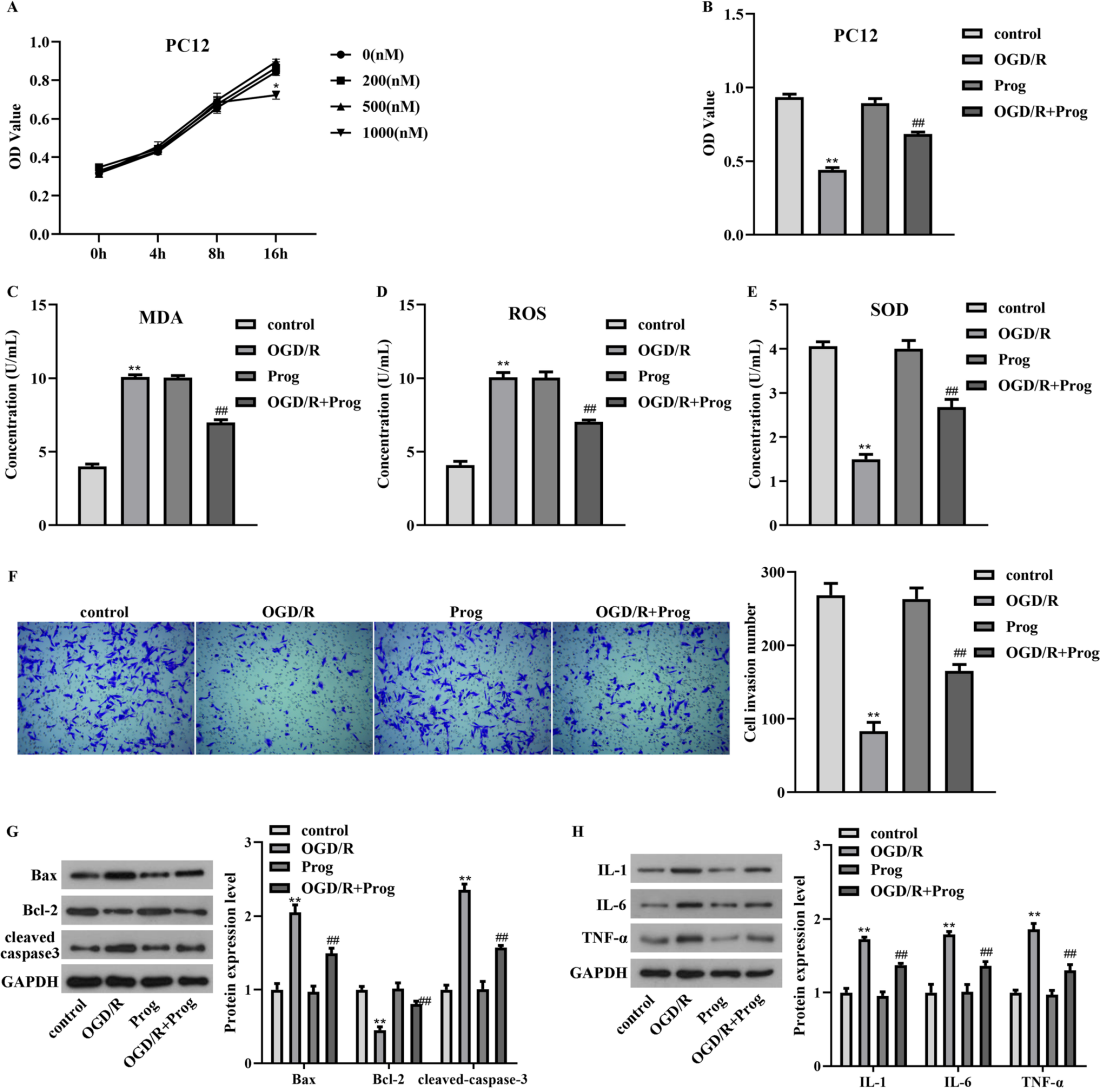
The protective effects of progesterone on PC12 cells have been demonstrated under OGD/R stimulation. A. The working concentration of progesterone has been identified. B. The effect of progesterone on PC12 cells viability has been revealed by CCK-8 assay. C-E. The levels of MDA, ROS, and SOD in PC12 cells have been detected by ELISA kits after the co-treatment of progesterone and OGD/R. F. The invasion number of PC12 cells was examined by transwell assay after the co-treatment of progesterone and OGD/R. G-H. The protein levels of Bax, Bcl-2, cleaved caspase3, IL-1, IL-6, and TNF-α in PC12 cells have been detected by western blot after the co-treatment of progesterone and OGD/R. **p < 0.01 vs. control, ##*p* < 0.01 vs. OGD/R

**Fig. 2:**
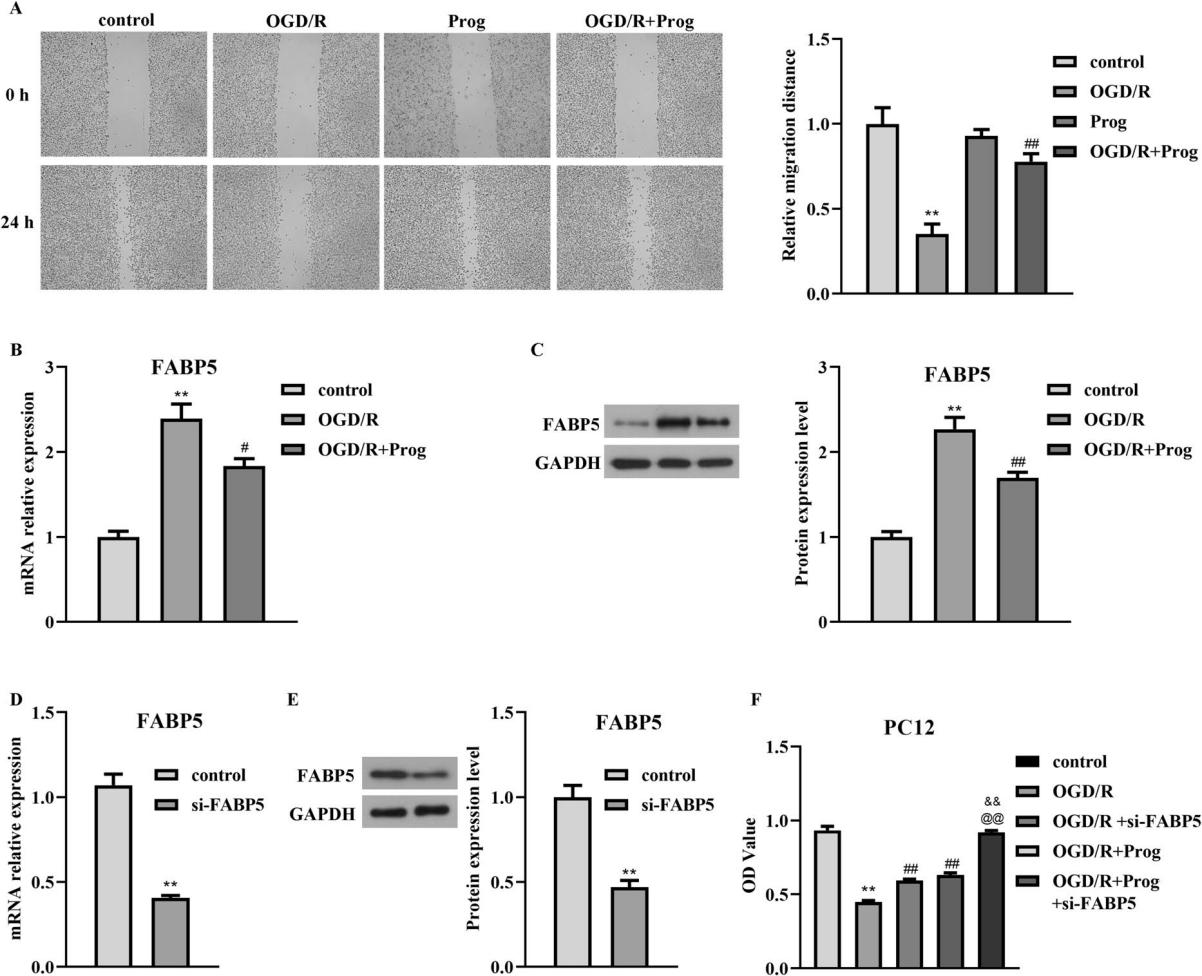
Progesterone affects the migration ability of PC12 cells and regulates FABP5 expression. A. The migration distance of PC12 cells was examined by wound healing assay after the co-treatment of progesterone and OGD/R. B-C. The mRNA and protein levels of FABP5 were examined by qPCR and western blot assays under OGD/R condition. D-E. The mRNA and protein levels of FABP5 were examined by qPCR and western blot assays after si-FABP5 treatment. F. The effect of progesterone and FABP5 on PC12 cells viability has been revealed by CCK-8 assay

To investigate the function of progesterone on OGD/R-induced PC12 cells, we first selected the effective working concentration of progesterone. Under normal condition, 0–500 nM progesterone has no obvious effect on PC12 cells viability. However, 1000 nM progesterone treatment significantly decreased the viability of PC12 cells (Fig. 1A, *p*  = 0.0169, F = 35.082). Therefore, 500 nM progesterone was selected for the subsequent research. After OGD/R stimulation, the OD values of PC12 cells decreased (Fig. 1B, *p*  < 0.01, F = 342.172), the production of MDA and ROS increased (Fig. 1C, *p*  < 0.01, F = 901.084; Fig. 1D, *p*  < 0.01, F = 291.538), the concentration of SOD decreased (Fig. 1E, *p*  < 0.01, F = 202.015), the invasion number of PC12 cells decreased (Fig. 1F, *p*  < 0.01, F = 132.399), the protein patterns of pro-apoptotic genes Bax, cleaved caspase3 increased, the protein patterns of anti-apoptotic gene Bcl-2 decreased (Fig. 1G), and the protein patterns of pro-inflammatory genes IL-1, IL-6 and TNF-α was also increased (Fig. 1H). Moreover, the relative migratory distance of PC12 cells reduced after OGD/R treatment (Fig. 2A, p  < 0.01, F = 63.034). However, with progesterone stimulation, the above injury of PC12 cells induced by OGD/R was diminished and tended to the normal level (Figs. 1B-H and 2A). Thus, the protective effects of progesterone on OGD/R-caused PC12 cells were demonstrated.

### FABP5 was identified as a specific target of progesterone

Information from CTD suggested that FABP5 was regarded as a target of progesterone. According to the prediction, FABP5 expression was measured in OGD/R-induced PC12 cells. Data from Fig. 2B-C showed that the expression patterns of FABP5 remarkably increased in OGD/R-induced PC12 cells (Fig. 2B, *p*  < 0.01, F = 108.001; Fig. 2C, *p*  < 0.01, F = 128.371), however, progesterone treatment suppressed the expression of FABP5. These results insinuated that FABP5 was a potential target of progesterone. To further detect the function of progesterone/FABP5 in OGD/R-induced PC12 cells, we used small interference RNA technique to knock down FABP5. As presented in Fig. 2D-E, the levels of FABP5 reduced after si-FABP5 treatment (Fig. 2D, *p*  < 0.01, F = 23.48; Fig. 2E, *p*  < 0.01, F = 2.862).

### Progesterone exerts protective effects on PC12 cells under OGD/R condition by targeting FABP5

**Fig. 3:**
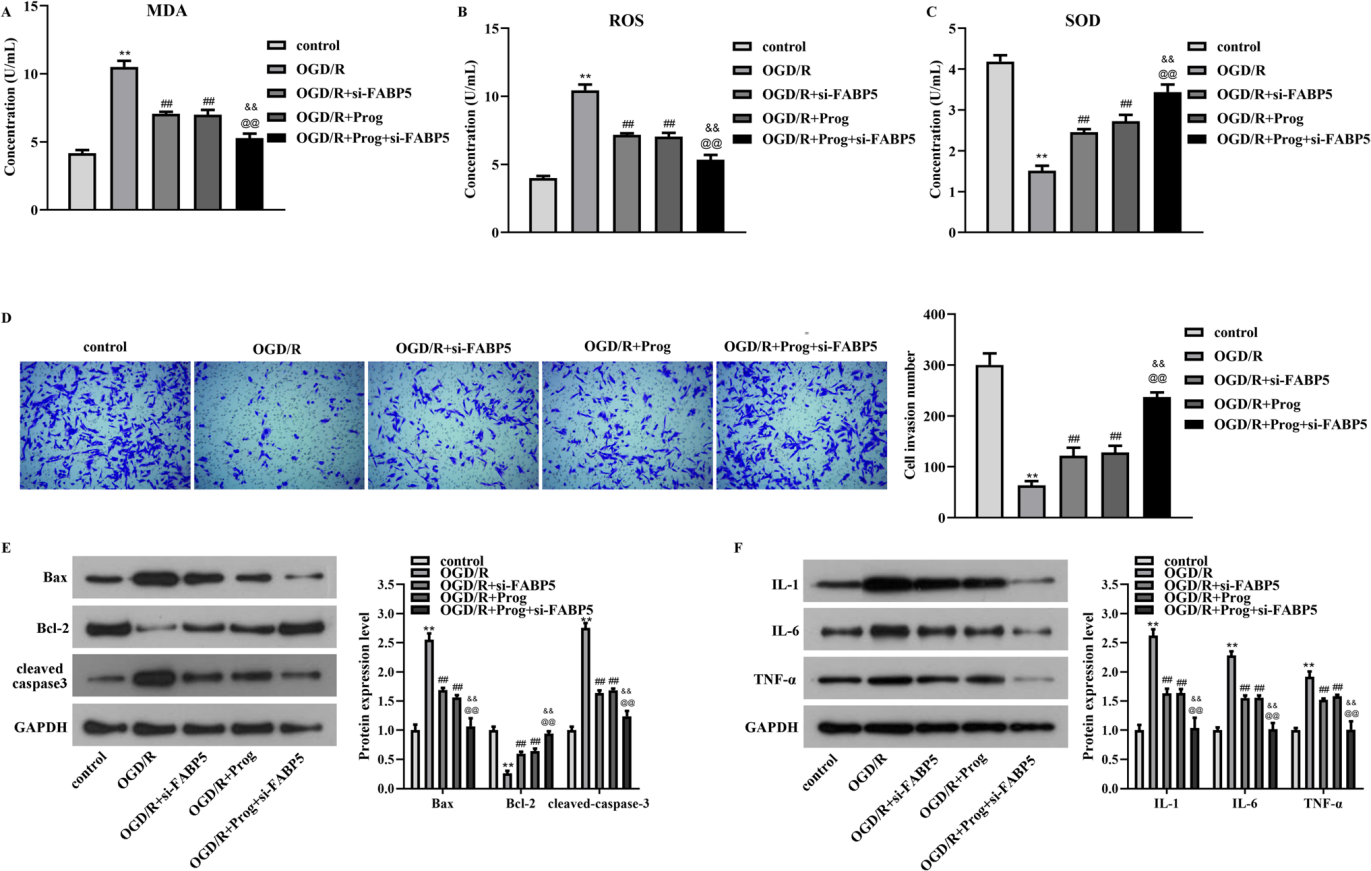
The beneficial effects of progesterone on OGD/R-induced PC12 cells were realized by targeting FABP5. A-C. The levels of MDA, ROS, and SOD in OGD/R-induced PC12 cells have been detected by ELISA kits after the co-treatment of progesterone and si-FABP5. D. The invasion number of PC12 cells was examined by transwell assay after the co-treatment of progesterone and si-FABP5 under OGD/R stimulation. E–F. The protein levels of Bax, Bcl-2, cleaved caspase3, IL-1, IL-6, and TNF-α in PC12 cells have been detected by western blot after the co-treatment of progesterone and si-FABP5 under OGD/R stimulation. **p < 0.01 vs. control, ##*p* < 0.01 vs. OGD/R, @@*p* < 0.01 vs. OGD/R + si-FABP5, & &*p* < 0.01 vs. OGD/R + Prog

**Fig. 4:**
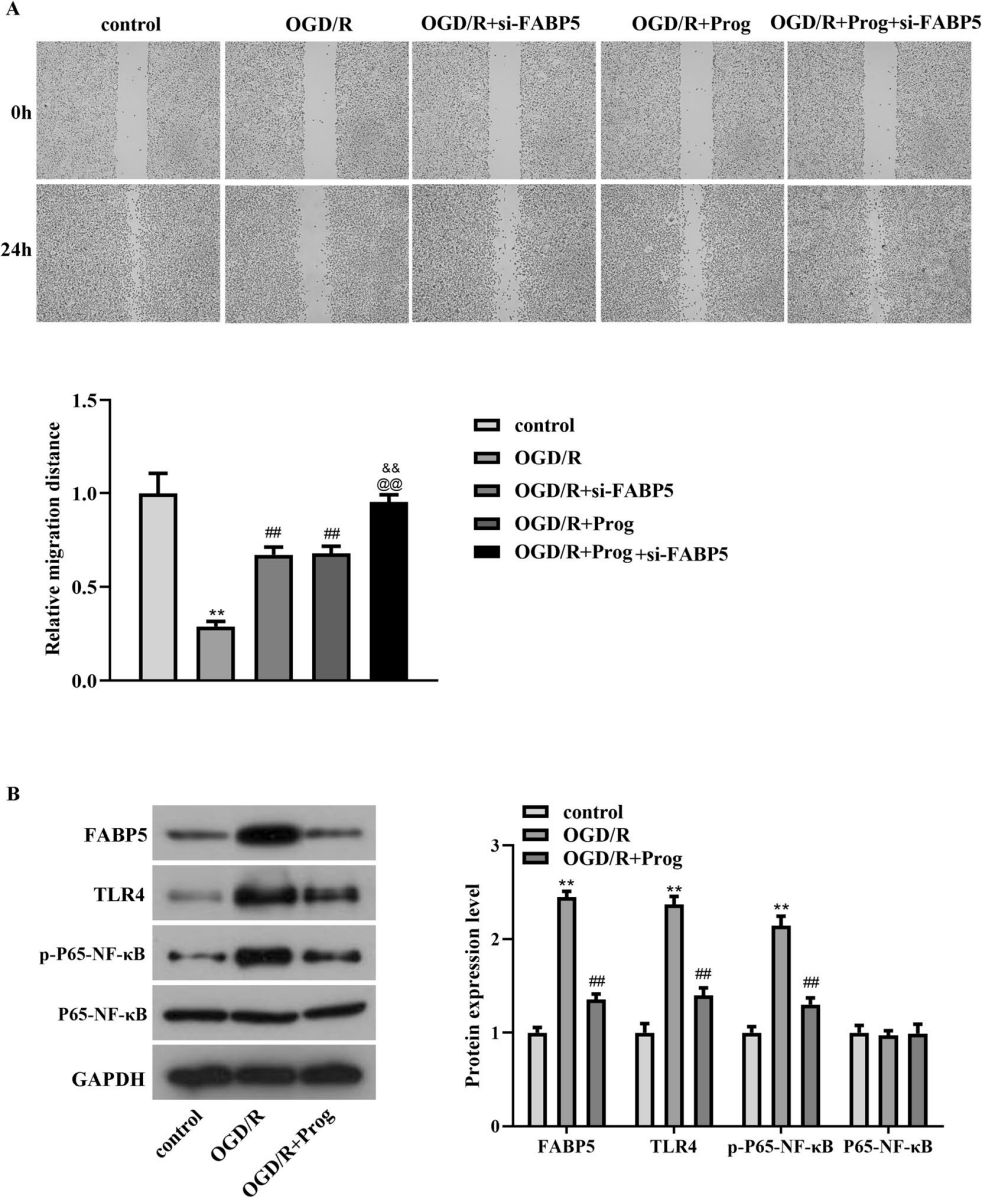
The beneficial effects of progesterone/FABP5 on PC12 cells were realized by regulating TLR4/NF-κB pathway. A. The migration distance of PC12 cells was examined by wound healing assay after the co-treatment of progesterone and si-FABP5 under OGD/R stimulation, **p < 0.01 vs. control, ##*p* < 0.01 vs. OGD/R, @@*p* < 0.01 vs. OGD/R + si-FABP5, & &*p* < 0.01 vs. OGD/R + Prog. B. The protein levels of FABP5, TLR4, p-P65 NF-κB, and P65 NF-κB in PC12 cells have been detected by western blot under OGD/R stimulation, **p < 0.01 vs. control, ##*p* < 0.01 vs. OGD/R

Subsequently, the OD value and the concentrations of MDA, ROS and SOD in PC12 cells were measured after progesterone or/and si-FABP5 treatment under OGD/R condition. The data from Fig. 2F (*p* < 0.01, F = 519.996) showed that the decrease in OD values caused by OGD/R in PC12 cells could be alleviated by treatment with progesterone or si-FABP5. Moreover, the co-treatment of progesterone and si-FABP5 led to more significant remission of PC12 cells viability. Results from Fig. 3A-C showed that the augment of MDA and ROS, and the reduction of SOD induced by OGD/R in PC12 cells could be alleviated by stimulation with progesterone or si-FABP5, which were more obvious after the co-treatment of progesterone and si-FABP5 (Fig. 3A, *p*  < 0.01, F = 165.016; Fig. 3B, *p*  < 0.01, F = 206.763; Fig. 3C, *p*   < 0.01, F = 149.184). Analysis from Fig. 3D (*p* < 0.01, F = 128.459) showed that progesterone or si-FABP5 treatment could alleviate the decrease in the number of PC12 cells invasion caused by OGD/R, and the effects of combined treatment with progesterone and si-FABP5 were more obvious. In PC12 cells, the protein expression of Bax and cleaved caspase3 was increased and Bcl-2 protein expression was decreased induced by OGD/R, which could be suppressed by progesterone or si-FABP5 treatment alone or in combination (Fig. 3E). Results from Fig. 3F showed that the elevated protein expression of IL-1, IL-6 and TNF-α caused by OGD/R could be suppressed by progesterone or si-FABP5 treatment alone or in combination. Moreover, results from wound healing assay suggested that the reduction of the relative migration distance of PC12 cells induced by OGD/R stimulation was mitigated by the addition of progesterone or si-FABP5, which were more obvious after the co-treatment of progesterone and si-FABP5 (Fig. 4A, *p*  < 0.01, F = 72.688).

### The beneficial effects of progesterone/FABP5 on PC12 cells were realized by regulating TLR4/NF-κB pathway

Previous study suggested that progesterone administration could inhibit TLR4/NF-κB signaling pathway in the rat brain following subarachnoid hemorrhage(Wang et al. 2011). In our study, the data from Fig. 4B showed that the patterns of FABP5, TLR4 and p-P65 NF-κB were obviously elevated under OGD/R treatment in PC12 cells. However, progesterone treatment significantly diminished the patterns of FABP5, TLR4 and p-P65 NF-κB in OGD/R caused PC12 cells. Therefore, our results suggested that progesterone may ameliorate OGD/R-caused damage of PC12 cells by inactivating FABP5/TLR4/NF-κB pathway.

## Discussion

It has been presented that progesterone possesses a therapeutic function in central nervous system diseases, including cerebral stroke. Herein, we carried out a study to explore the molecular mechanism of progesterone in OGD/R-induced PC12 cells injuries. Our findings revealed that the administration of progesterone could alleviate OGD/R-induced PC12 cells injury by augmenting the growth and migration, and diminishing inflammation, apoptosis and oxidative stress. Moreover, FABP5 was identified as a target of progesterone, and knocked down of FABP5 could strengthen the beneficial function of progesterone on OGD/R-caused PC12 cells by regulating TLR4/NF-κB pathway.

Progesterone is a neuroprotective and anti-inflammatory factor in the nervous system (Nicola et al. 2013). However, the detailed mechanism of progesterone has not yet been elucidated. In our study, we discovered that.

Recently, a study demonstrated that FABP5 has been obviously increased in the mouse brain after I/R stimulation (Guo and Kawahata 2021). In addition, FABP5 has been reported to cause cell death under oxidative stress in glial cells (Cheng and Kawahata 2020). Consistent with the published report (Guo and Kawahata 2021), our finding revealed that FABP5 was obviously augmented in PC12 cells after OGD/R stimulation. Moreover, under OGD/R condition, knocked down of FABP5 could strengthen PC12 cells viability and migration abilities, and attenuate the inflammation, oxidative stress and apoptosis of PC12 cells. Furthermore, our results suggested that FABP5 level was diminished by progesterone in OGD/R-induced PC12 cells, which was consistent with the reduction of FABP5 expression reported in CTD website.

Inflammatory factors have been found to be important contributing mediators in cerebral ischemic injury (Liu et al. 2015). Moreover, a recent study suggested that TLR4 could contribute to I/R induced inflammatory response (Zhang and Song 2020). Nuclear factor-kappa B (NF-κB) has been reported to take part in some cellular behaviors such as inflammation, cell apoptosis and immune response (Zhu et al. 2018). Additionally, NF-κB has been found to modulate the generation of numerous pro-inflammatory factors in neuronal cells, such as IL-1β, IL-6 and TNF-α (Hwang et al. 2015). All the background emphasized the significance of TLR4/ NF-κB pathway in inflammatory-related diseases. In our study, we discovered that TLR4 and p-P65 NF-κB expression levels were obviously augmented in PC12 cells-triggered by OGD/R. Progesterone has been reported to alleviate temporomandibular joint inflammation via suppression of NF-κB pathway in ovariectomized rats (Xue et al. 2017). Moreover, progesterone has been found to downregulate the cortical levels of these factors related to TLR4/NF-κB signaling pathway in the early brain injury (Wang et al. 2011). Herein, our results suggested that the increased levels of TLR4 and p-P65 NF-κB in OGD/R-triggered PC12 cells have been suppressed after progesterone administration.

Although we have made some discoveries, there are still some deficiencies in the article that need to be pointed out. First, our results were achieved just based on functional experiments on PC12 cells in vitro, which need to be further confirmed in animals in the future. Second, the function of progesterone in OGD/R-induced PC12 cells is not only depended on FABP/TLR4/NF-κB pathway, and more molecular mechanisms need to be explored.

In summary, we discovered that the supplementation of progesterone could mitigate the damage of PC12 cells caused by OGD/R, whereas the increased expression of FABP5 in OGD/R-induced PC12 cells can be suppressed after the administration of progesterone, and knocked down of FABP5 consolidated the protective function of progesterone on OGD/R-triggered PC12 cells; additionally, the TLR4/NF-κB pathway related proteins were influenced by progesterone.

### Supplementary Information

Below is the link to the electronic supplementary material.

Fig. S1 (PNG 1500 kb)

High resolution (TIF 17077 kb)

Fig. S2 (PNG 2726 kb)

High resolution (TIF 28413 kb)

## Data Availability

The datasets used during the study are available from the corresponding author on reasonable request.
