# Supplementary figures and images for: Progesterone improved the behavior of PC12 cells under OGD/R by reducing FABP5 expression and inhibiting TLR4/NF-κB signaling pathway
Source: J Bioenerg Biomembr. 2023 Dec 18;56(2):117–24. doi: 10.1007/s10863-023-09998-z (PMC10995011; doi:10.1007/s10863-023-09998-z)

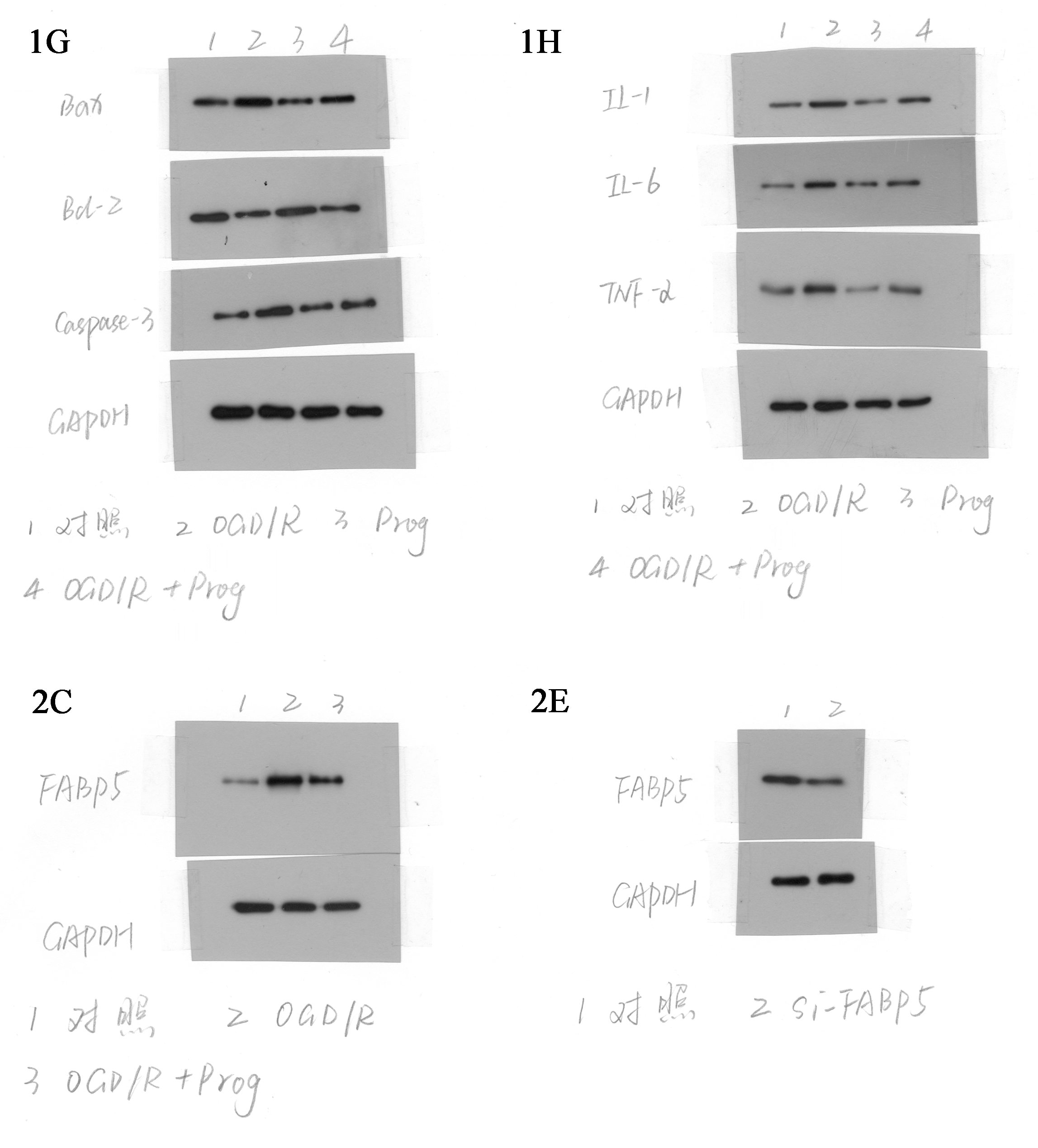

Supplement: Supplementary file 1 — (PNG 1500 kb) [file 10863_2023_9998_Fig5_ESM.png]

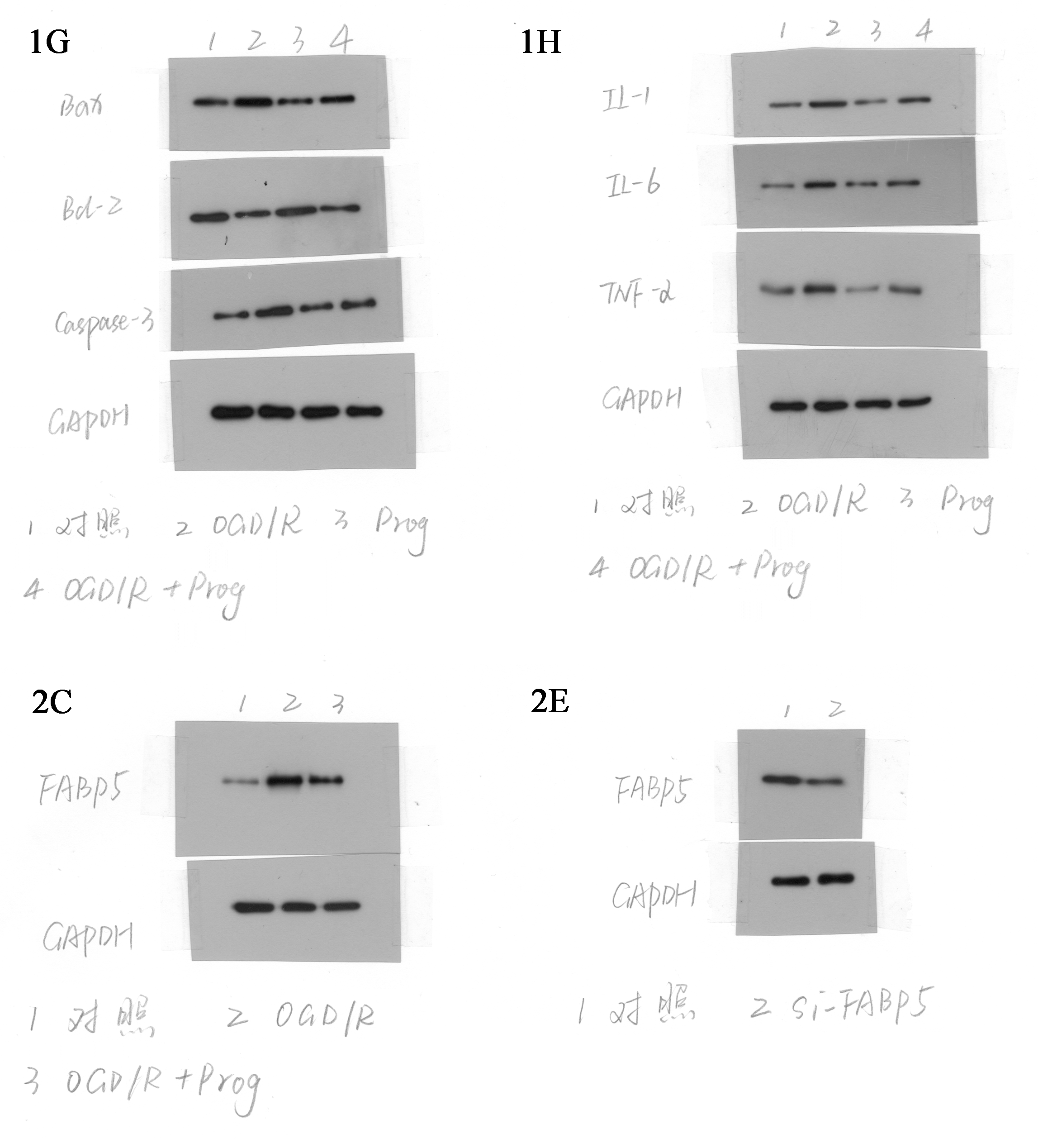

Supplement: Supplementary file 2 — High resolution (TIF 17077 kb) [file 10863_2023_9998_MOESM1_ESM.tif]

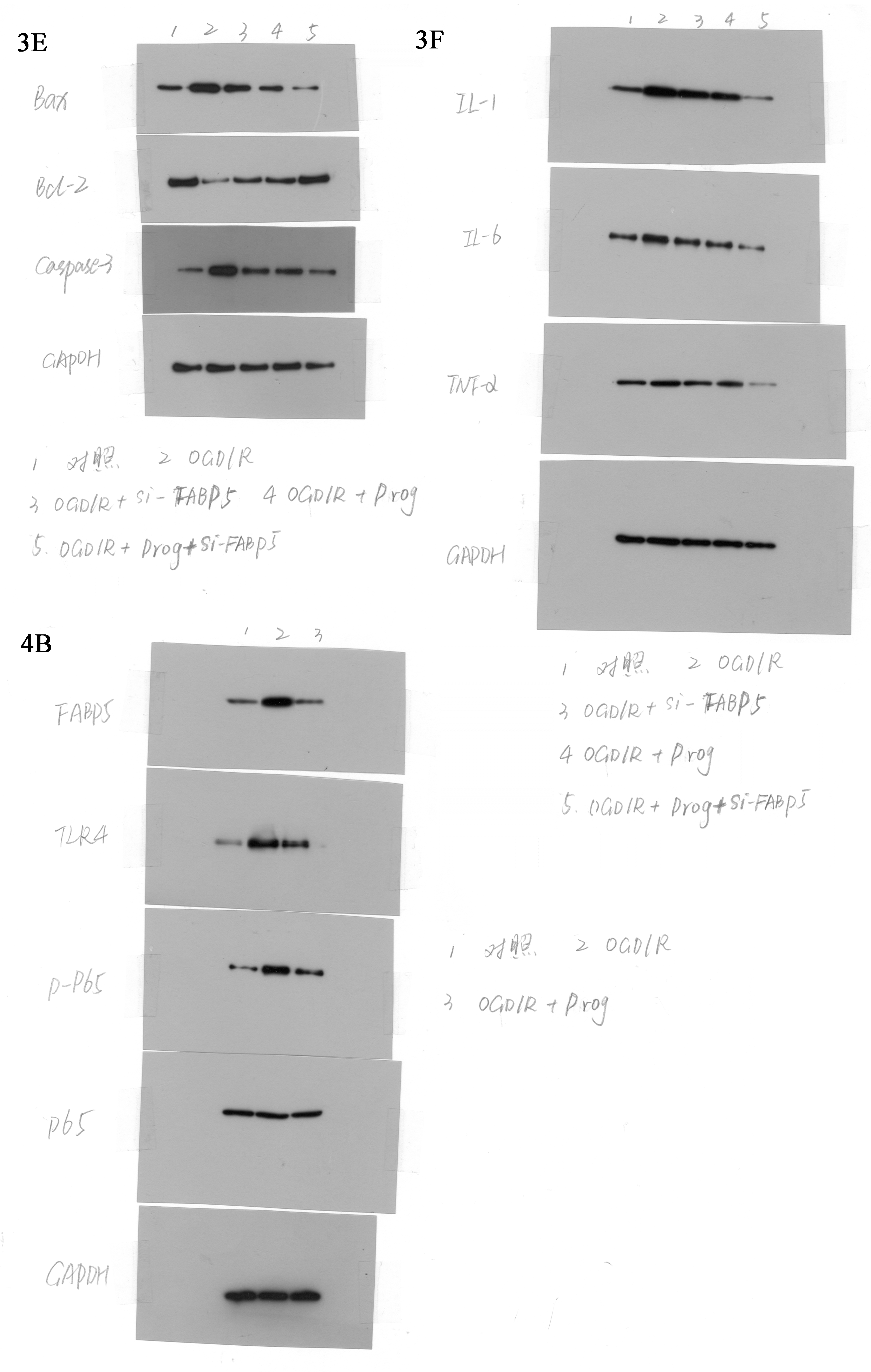

Supplement: Supplementary file 3 — (PNG 2726 kb) [file 10863_2023_9998_Fig6_ESM.png]

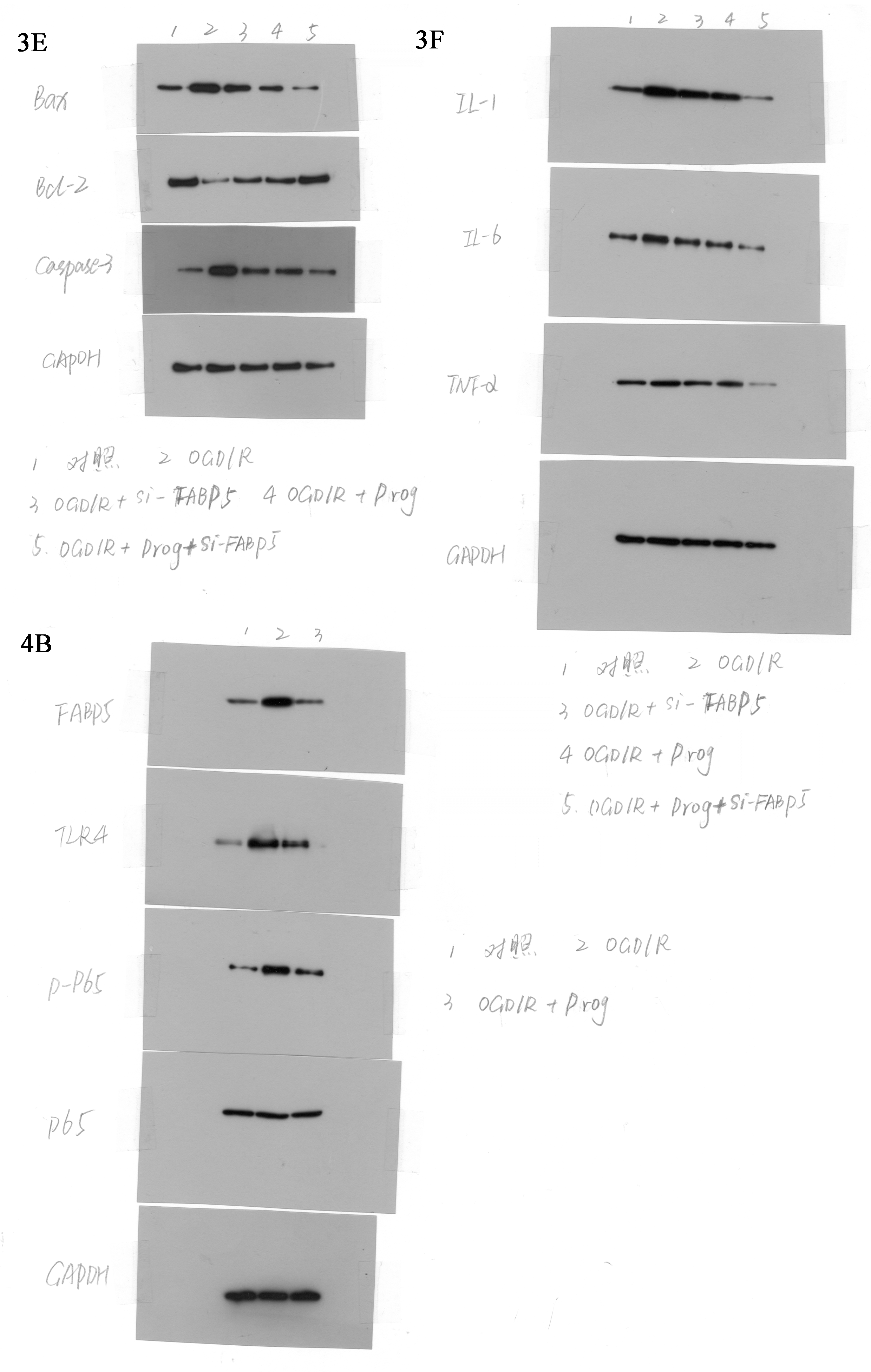

Supplement: Supplementary file 4 — High resolution (TIF 28413 kb) [file 10863_2023_9998_MOESM2_ESM.tif]
